# Cell-free microRNAs: potential biomarkers in need of standardized reporting

**DOI:** 10.3389/fgene.2013.00056

**Published:** 2013-04-19

**Authors:** Michaela B. Kirschner, Nico van Zandwijk, Glen Reid

**Affiliations:** Asbestos Diseases Research Institute, University of SydneySydney, NSW, Australia

**Keywords:** cell-free microRNA, isolation, quantification, reporting, standard

## Abstract

MicroRNAs are abundantly present and surprisingly stable in multiple biological fluids. These findings have been followed by numerous reverse transcription real-time quantitative PCR (RT-qPCR)-based reports revealing the clinical potential of using microRNA levels in body fluids as a biomarker of disease. Despite a rapidly increasing body of literature, the field has failed to adopt a set of standardized criteria for reporting the methodology used in the quantification of cell-free microRNAs. Not only do many studies based on RT-qPCR fail to address the Minimum Information for Publication of Quantitative Real-Time PCR Experiments (MIQE) criteria but frequently there is also a distinct lack of detail in descriptions of sample source and RNA isolation. As a direct result, it is often impossible to compare the results of different studies, which in turn, hinders progress in the field. To address this point, we propose a simple set of criteria to be used in conjunction with MIQE to reveal the true potential of cell-free microRNAs as biomarkers.

## INTRODUCTION

In recent years, a number of studies have shown that cell-free microRNAs are readily detectable in body fluids, such as plasma or serum. The surprising stability of cell-free microRNAs in these body fluids has been attributed to encapsulation of microRNAs in microvesicles such as exosomes (Cortez and Calin, 2009; Kosaka et al., 2010), and association with protein complexes, like high density lipoproteins (Vickers et al., 2011) and argonaute 2 (Arroyo et al., 2011; Turchinovich et al., 2011). These findings led to numerous studies aiming at the identification of microRNAs, mainly in plasma and serum, as potential diagnostic and/or prognostic markers for a variety of diseases (Reid et al., 2011; Creemers et al., 2012; Mo et al., 2012). However, although most studies are using reverse transcription real-time quantitative PCR (RT-qPCR) for detection of microRNAs, there is still a lack of consensus regarding isolation procedures, optimal quantification methods, reference genes, and quality control of samples. In addition, most studies fail to provide sufficient detail on the methods used for RNA isolation, quality control of samples, and do not report according to Minimum Information for Publication of Quantitative Real-Time PCR Experiments (MIQE) criteria (Bustin et al., 2009). As a result, comparisons between studies from different laboratories are almost impossible, and it is not surprising that different studies using profiling strategies to identify plasma/serum biomarkers for the same disease fail to identify the same microRNAs.

In order to overcome these problems, we propose a simple set of reporting criteria to be used in conjunction with MIQE reporting guidelines (Bustin et al., 2009). The following sections outline the steps undertaken to quantify cell-free microRNAs together with important aspects to be considered in each step. We limit the microRNA quantification section to RT-qPCR as this is the most commonly used method for microRNA detection, and for simplicity we focus on plasma and serum, but many aspects will apply equally to other quantification methods (such as microarray) or other biological fluids. Finally, we provide lists of important points to be included when reporting investigation of cell-free microRNAs.

## SAMPLE COLLECTION

The first critical step in the process of assessing levels of cell-free microRNAs in plasma and serum is the collection and handling of the sample. Both serum and plasma are suitable for assessment of cell-free microRNAs, but when using plasma samples ethylenediaminetetraacetic acid (EDTA) or citrate are the preferred anticoagulants as heparin is known to inhibit both reverse transcription and qPCR steps (Kroh et al., 2010). The use of more than one type of collection tube within a study is generally discouraged as the resulting microRNA profiles can vary between different tubes, making comparisons inaccurate (Kroh et al., 2010). Reports on cell-free microRNAs should therefore always include details regarding the blood collection procedure, type of blood collection tube, and processing of the sample.

Essential information to be provided:

–Collection site, caliber, and type of needle/cannula
–Type and size of collection tube (including manufacturer)
–Time between collection and further processing
–In case of serum, time allowed for clotting
–Conditions of sample processing (centrifugation speed and time)
–Storage condition of samples

## QUALITY CONTROL OF SAMPLES

One of the most critical points in the process of quantifying cell-free microRNAs is that of quality assurance and quality control (QA/QC). While being neglected in the early stages of cell-free microRNA research, a small number of studies have now shown that the contamination with microRNAs released from circulating blood cells represents one of the major sources of microRNAs detectable in plasma or serum (Kirschner et al., 2011; McDonald et al., 2011; Pritchard et al., 2012). The most common source of blood cell derived microRNAs are red blood cells (RBCs) and two studies have shown that hemolysis occurring during sample collection or processing can significantly alter the levels of certain RBC-enriched microRNAs, such as miR-16 and miR-451 (Kirschner et al., 2011; McDonald et al., 2011). In addition, microRNAs released from platelets can also alter the microRNA profile (Pritchard et al., 2012). These changes caused by rupturing of blood cells result in variation in microRNA levels independent of the presence or absence of a certain disease state. While not yet investigated in detail, one can confidently assume that other microRNAs, including those previously being proposed as potential biomarkers for disease can be affected in a similar way to, e.g., miR-16 and miR-451. Measuring the degree of hemolysis, a major source of blood cell microRNAs in the fluid component, can be easily achieved by assessing the presence of free hemoglobin through simple measurement of the absorbance at 414 nm (the absorbance maximum of free hemoglobin) in the initial plasma or serum sample using a standard spectrophotometer (Wong et al., 2006). Data on the degree of hemolysis of samples should always be provided, and applying a cut-off level of free hemoglobin to determine if samples are usable for further analysis, such as an OD_414_ of 0.2 as we have suggested (Kirschner et al., 2011), should always be considered. In addition, like for formalin-fixed paraffin-embedded (FFPE) tissue samples (Szafranska et al., 2008), the age of a sample will certainly play a role in respect to the quality of RNA that can be obtained (Grasedieck et al., 2012). Both storage condition and sample age should therefore be provided.

## RNA ISOLATION

**FIGURE 1:**
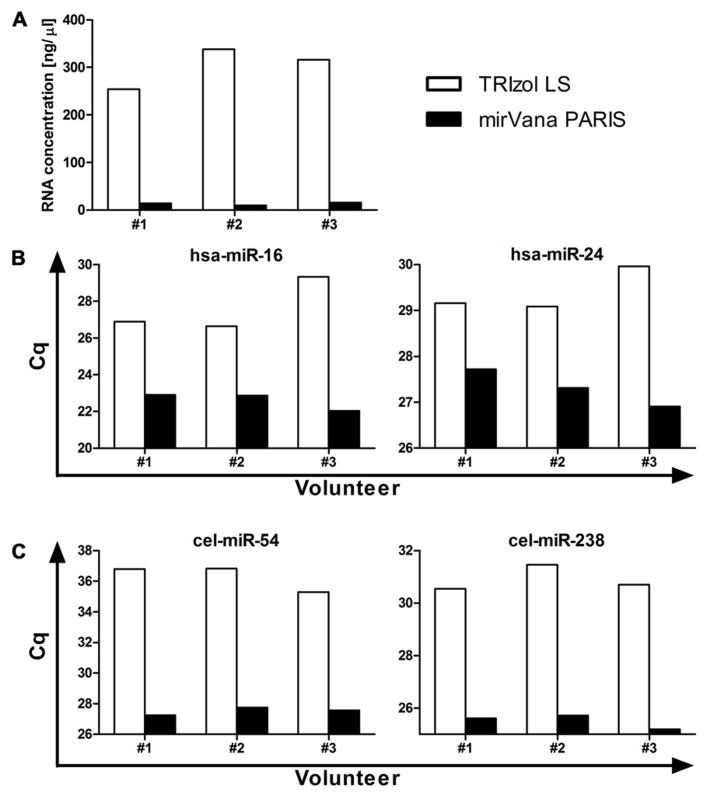
**Comparison between TRIzol LS and mirVana PARIS microRNA isolation efficiency.** Total RNA was isolated from 500 μl plasma from three healthy volunteers and total RNA concentration **(A)**, levels of moderately to highly abundant endogenous microRNAs **(B)**, and of exogenous spike-ins **(C)** were assessed. RT-qPCR data are presented as raw Cq values.

The isolation of RNA represents another critical step in order to obtain a sample of acceptable quality for further investigation. The most commonly used methods for RNA isolation from body fluids are (i) column-based purification with mostly mirVana PARIS and Qiagen (miRNeasy Serum/Plasma Kit) or (ii) non-column purification using reagents such as TRIzol LS or QIAzol. While no study has yet shown a direct comparison between column-based and non-column purification, a study in cell lines suggests that care must be taken regarding the choice of isolation method. Kim et al. (2012) have shown that when isolating RNA from cells using a non-column method the number of cells seems to not only affect the efficiency of RNA isolation in general, but also result in a selective loss of microRNAs with low guanine–cytosine (GC) content when isolating from a small cell number, hence when handling samples with low RNA concentration. Plasma and serum are low RNA concentration samples, raising the possibility that preferential isolation of a subset of microRNAs might occur too when using non-column purification. In addition, data from our own studies generally comparing TRIzol LS and mirVana PARIS isolation efficiencies have shown that while TRIzol LS purification results in higher levels of total RNA recovered from plasma samples (**Figure 1A**), mirVana PARIS is superior in isolating small RNAs as shown for both moderately to highly abundant endogenous microRNAs (**Figure 1B**), and exogenous spike-ins (**Figure 1C**). Nevertheless, as no formal comparison for larger numbers of microRNAs has been performed so far for plasma and serum, no firm conclusions can be drawn regarding the best isolation method for these sample types.

Regardless of the relative merits of the various options for RNA isolation, studies investigating cell-free microRNAs should always provide sufficient details on the isolation method used, and these should be:

–Sample volume from which RNA was isolated
–Reagent or kit used for RNA isolation
–Whether a carrier (glycogen, tRNA, etc.) was used, the concentration of any such reagent and at which step of the isolation they have been added
–Whether and in which concentration any spike-ins such as synthetic *C. elegans* microRNAs have been used and at which step of the isolation process those have been added
–The volume used for resuspension or elution of the final RNA sample.

## RNA QUANTIFICATION

Quantification of RNA isolated from dilute samples such as plasma/serum can be very difficult (Kroh et al., 2010). Even when using a carrier to enhance the isolation efficiency, attempts to measure RNA concentration by standard spectrophotometry often fail due to the detection limit of this method (generally around 4–10 ng/μl). Alternatively fluorometric quantitation can be used, however, especially when using serum the concentration of the obtained samples can still be too low for accurate quantitation. Using larger sample input into the isolation process may overcome this problem. However, large amounts of sample are not always available and most studies have shown that microRNAs are readily detectable when isolating from 500 μl or less of plasma/serum (Kroh et al., 2010).

As a consequence of these difficulties in quantifying the RNA concentration of dilute solutions, it is in many cases not possible to use equal amounts (e.g., nanogram) of template RNA for each sample in the subsequent reverse transcription step. Often the only alternative is to use equal input volume of RNA (Mitchell et al., 2008), but this approach requires the availability of an alternative normalization method to account for differences in input RNA concentration. The most commonly used approaches here are the use of an exogenous spike-in microRNA of known quantity (Mitchell et al., 2008) or the use of endogenous control microRNAs which do not vary significantly between samples obtained from study and control subjects (Kirschner et al., 2011). However, both these strategies are associated with further problems which are discussed in more detail below.

## REVERSE TRANSCRIPTION REAL-TIME QUANTITATIVE PCR

A number of different technologies are available for RT-qPCR quantification of microRNAs. The most commonly used technologies are the microRNA-specific stem-loop reverse transcription in combination with TaqMan qPCR primer/probe assays (Life Technologies) or the combination of poly-A-tailing in the reverse transcription step followed by either locked-nucleic-acid (LNA)-enhanced forward and reverse primers (Exiqon) or standard SYBR green forward and reverse primers (Qiagen). Each technology provides advantages and disadvantages, but the superiority of one system over the other has not been proven (Zampetaki and Mayr, 2012). When reporting on RT-qPCR for cell-free microRNAs it is therefore essential to provide sufficient detail (following the MIQE guidelines; Bustin et al., 2009) to allow the reader to understand and reproduce published data using the same technology.

The minimum details that should be provided are:

–Primer sequences or exact assay IDs/catalog numbers when using proprietary assays
–Amount of RNA used as input into reverse transcription, or volume of eluate
–Reverse transcription procedure (poly-A-tailing or stem-loop) including all reagents and concentrations used, and reaction conditions (even if as per manufacturer)
–Details of instrument used to perform reverse transcription
–Whether preamplification was done, and if so how
–Dilution of cDNA and amount of cDNA used in qPCR
–All reagents and the concentrations used for qPCR as well as reaction conditions
–Details on instrument used to run qPCR and methods used to determine threshold (details on software used to analyze qPCR data/determine threshold)

## NORMALIZATION OF RT-qPCR DATA

The final and perhaps most critical step when quantifying cell-free microRNAs is the quantification or normalization method used. With the amount of RNA isolated from plasma or serum being very low and even with the use of carrier reagents during RNA isolation often below the threshold of detection (at least for standard spectrophotometry), it is frequently not possible to normalize the concentration of RNA used for reverse transcription. Equal volumes of input RNA are therefore often used instead of a fixed concentration; however this approach introduces the variable associated with comparing samples of unknown RNA concentration.

The major strategies proposed to overcome these problems are (i) the use of endogenous control microRNAs for normalization (Kirschner et al., 2011), (ii) the use of known quantities of exogenous microRNAs (mainly *C. elegans* miRs) that are spiked-in to compensate for differences in isolation efficiency (Mitchell et al., 2008; Kroh et al., 2010), and (iii) absolute quantification based on standard curves generated from synthetic microRNAs.

While an absolute quantification would most likely be the preferred measurement for a clinical setting, the variability in RNA input into the reverse transcription still leaves the problem of normalization between different samples.

Measurement of microRNAs spiked-in during the process of RNA isolation has the potential to account for the variability in initial RNA concentration. However, these spiked-in oligos do not account for variability caused by ruptured blood cells (Kirschner et al., 2011; McDonald et al., 2011; Pritchard et al., 2012). As discussed above, the quality of a sample, in particular the degree of hemolysis can have substantial impact on the levels of microRNAs measured in plasma or serum and should therefore always be taken into consideration. Spiked-in microRNAs are not suited to be used as a second quality control step, like miR-16 or miR-451 are, and one should therefore be careful when using only those as normalization controls for qPCR data. Nevertheless, if spiked-in oligos are used then more information should be provided, including some indication of recovery (%) and variation between samples.

Endogenous microRNAs such as miR-16 and miR-451 are among the most abundant microRNAs present in plasma. These microRNAs represent suitable candidates for normalization of qPCR data. However, as it is not known yet if other microRNAs are also affected by hemolysis, one cannot use miR-16 and miR-451 to adjust for varying degrees of hemolysis in different samples. A detailed analysis of the microRNA profiles in non-hemolyzed and hemolyzed samples is required to determine which microRNAs are affected by rupturing of RBCs. This exercise may help us to define subsets of microRNAs that are affected by hemolysis in the same way, thereby allowing a combination of endogenous control and biomarkers microRNA(s) to be selected. Independent of the outstanding problems associated with the sample quality’s effect on cell-free microRNAs, an additional problem with many studies currently being published is a lack of detail regarding the exact ways of normalization and calculation of relative abundance levels of microRNAs.

Studies reporting on cell-free microRNAs should provide the following details:

–Whether a fixed volume or a fixed RNA concentration was used as input into reverse transcription
–Whether a spiked-in microRNA(s) was used, and if so how many and the exact concentration added during the isolation procedure
–If spiked-in microRNAs were used, at least the average recovery of those should be reported
–In case of normalization to a spiked-in microRNA or endogenous control, details on the normalization procedure should be provided (equations used)
–In case of reporting relative expression in one group as compared to another group, details on the calculations should be provided (2^-ΔΔCq^; Livak and Schmittgen, 2001; Schmittgen and Livak, 2008) is often not detailed enough – how exactly was ΔΔCq calculated?)
–Raw data should be provided

## CONCLUSION/PERSPECTIVE

**FIGURE 2:**
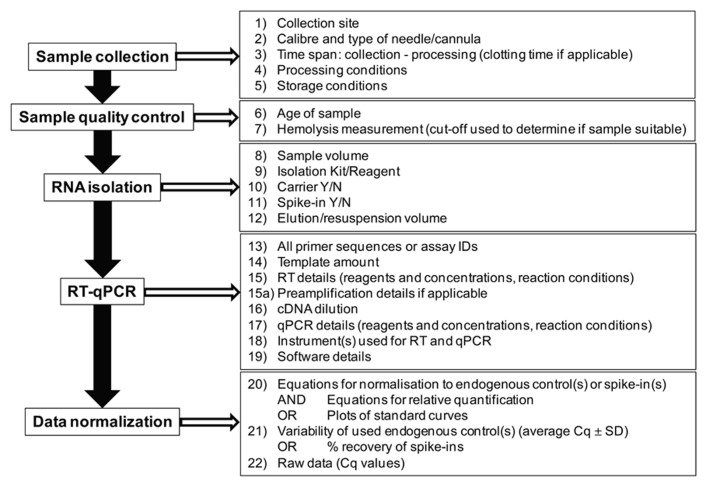
**Summary of details that should be provided when reporting on cell-free microRNAs**.

Since their discovery, cell-free microRNAs have been quickly introduced as potential biomarkers. However, it is important to bear in mind that the field of cell-free microRNA research is still in its infancy and that a lack of standardized reporting is making comparisons between studies as well as reproducibility of published data in independent sample series virtually impossible. While we have not solved all the problems associated with quantification of cell-free microRNAs, such as the most appropriate normalization strategies or whether samples with hemolysis can/should be used for analysis, the field would certainly benefit from a standardized way of reporting as summarized in **Figure 2**. Standardized reporting will allow researchers to better understand the data obtained in different laboratories and to use the same methods for potential follow-up or validation studies. Single discovery studies are unlikely to result in the discovery of novel biomarkers for clinical use and it is important to provide all the details of the methodology used to allow further validation by independent investigators. Only in this way the field is enabled to advance eventually leading to the implementation of novel microRNA-based diagnostic/prognostic tests.

## Conflict of Interest Statement

The authors declare that the research was conducted in the absence of any commercial or financial relationships that could be construed as a potential conflict of interest.
